# Microbiota and Lung Cancer. Opportunities and Challenges for Improving Immunotherapy Efficacy

**DOI:** 10.3389/fonc.2020.568939

**Published:** 2020-09-24

**Authors:** Maitane Ocáriz-Díez, Mara Cruellas, Marta Gascón, Rodrigo Lastra, Luis Martínez-Lostao, Ariel Ramírez-Labrada, José Ramón Paño, Andrea Sesma, Irene Torres, Alfonso Yubero, Julián Pardo, Dolores Isla, Eva M. Gálvez

**Affiliations:** ^1^Medical Oncology Department, Lozano Blesa University Clinical Hospital, Zaragoza, Spain; ^2^Instituto de Investigación Sanitaria Aragón (IIS Aragón), Zaragoza, Spain; ^3^Inmunology Department, Lozano Blesa University Clinical Hospital, Zaragoza, Spain; ^4^Department of Microbiology, Pediatrics, Radiology and Public Health, University of Zaragoza, Zaragoza, Spain; ^5^Aragon Nanoscience Institute, Zaragoza, Spain; ^6^Aragon Materials Science Institute, Zaragoza, Spain; ^7^Unidad de Nanotoxicología e Inmunotoxicología (UNATI), Fundación Instituto de Investigación Sanitaria Aragón (IIS Aragón), Biomedical Research Centre of Aragón (CIBA), Zaragoza, Spain; ^8^Infectious Diseases Department, Lozano Blesa University Clinical Hospital, Zaragoza, Spain; ^9^ARAID Foundation (IIS Aragón), Zaragoza, Spain; ^10^Microbiology, Preventive Medicine and Public Health Department, Medicine, University of Zaragoza, Zaragoza, Spain; ^11^Biomedical Research Center in Bioengineering, Biomaterials and Nanomedicine Network (CIBER-BBN), Madrid, Spain; ^12^Instituto de Carboquimica (ICB-Consejo Superior de Investigaciones Cientificas), Zaragoza, Spain

**Keywords:** microbiota, lung cancer, immunotherapy, lung microbiota, antibiotics

## Abstract

The advances in molecular biology and the emergence of Next Generation Sequencing (NGS) have revealed that microbiome composition is closely related with health and disease, including cancer. This relationship affects different levels of cancer such as development, progression, and response to treatment including immunotherapy. The efficacy of immune checkpoint inhibitors (ICIs) may be influenced by the concomitant use of antibiotics before, during or shortly after treatment with ICIs. Nevertheless, the linking mechanism between microbiote, host immunity and cancer is not clear and the role of microbiota manipulation and analyses in cancer management has not been clinically validated yet. Regarding the use of microbiome as biomarker to predict ICI efficacy it has been recently shown that the use of biochemical serum markers to monitor intestinal permeability and loss of barrier integrity, like citrulline, could be useful to monitor microbiota changes and predict ICI efficacy. There are still many unknowns about the role of these components, their relationship with the microbiota, with the use of antibiotics and the response to immunotherapy. The next challenge in microbiome research will be to identify individual microbial species that causally affect lung cancer phenotypes and response to ICI and disentangle the underlying mechanisms. Thus, further analyses in patients with lung cancer receiving treatment with ICIs and its correlation with the composition of the microbiota in different organs including the respiratory tract, peripheral blood and intestinal tract could be useful to predict the efficacy of ICIs and its modulation with antibiotic use.

## Introduction

Lung cancer is the most common cancer and the most frequent cause of cancer-related deaths worldwide both in men and women (1). Every year, more than 1.8 million people are diagnosed with lung cancer, accounting for 1.6 million deaths as a result of the disease. 5-year survival rates vary between 4 and 17% depending on stage and national differences (2). Non-small cell lung cancer (NSCLC) accounts for 80%−90% of lung cancers, while small cell lung cancer (SCLC) has been decreasing in frequency in many countries over the past two decades (1). Tobacco consumption represents one of the main risk factors for lung cancer due to the presence of carcinogens that promote cell transformation. However, other factors like chronic inflammatory immune-mediated responses, genetic predisposition or infections, like viral or more recently described bacterial, have been involved in lung carcinogenesis (3).

The clinical management of this neoplasm has undergone constant modifications, due to better knowledge of the biology of the tumor and the genomic profile of each patient, which allows a more accurate diagnosis and efficient treatment. During the last few years, new therapies directed against molecular targets involved in the regulation of the immune homeostasis and cancer immunity have been incorporated, particularly immune checkpoint inhibitors (ICIs), such as monoclonal antibodies against the Programmed Cell Death Protein 1/Ligand 1 (PD1/L1) axes or Cytotoxic T-Lymphocyte Antigen 4 (CTLA-4) (4). These molecules are critical negative regulators of the immune system that in physiological conditions prevent reactions against self-antigens avoiding autoimmune reactions. Some tumors have learnt to use these molecules to avoid immune attack and perpetuate in the host, and thus, under specific situations blocking ICs release these brakes and promote immune-mediated tumor elimination.

ICIs are effective in locally advanced unresectable NSCLC as consolidation after chemoradiotherapy (durvalumab) (5), in patients with pretreated metastatic disease (nivolumab, pembrolizumab, atezolizumab) (6–9) and also as first-line treatment, both as monotherapy (pembrolizumab) (10), in combination with chemotherapy (pembrolizumab, atezolizumab) (11, 12) or as double immunotherapy (anti-PD1 nivolumab/anti-CTLA4 ipilimumab) (13). Recently it has also been found to improve outcomes in metastatic SCLC as first line treatment in combination with chemotherapy (atezolizumab, durvalumab) (14, 15).

Despite these positive results, the major challenge that clinicians face is the identification of those patients that will benefit most from this therapy, including efficacy and low toxicity. At present, the most commonly used biomarker to predict ICIs efficacy, PDL1 expression, presents several limitations (16).

In addition to prevent unwanted reactions against self-antigens, ICIs are key molecules involved in the homeostatic regulation of the immune response against commensal microbiota. Thus, in recent years, microbiota has emerged as critical regulator of the efficacy and toxicity of ICIs in different types of cancer including lung. Here after a brief summary of the main experimental evidences relating microbiota with lung cancer development, we will discuss the relationship between the composition of lung microbiota and the efficacy of ICIs and the opportunities that this connection offers to exploit microbiome analyses and modification in the prediction of ICIs efficacy (17). Finally, we will present some of the main challenges that will be required to overcome in order to use microbiota modification as a therapeutic approach to enhance ICIs efficacy and safety.

## Microbiota and Tissue Homeostasis

The microbiota is emerging as a key regulator of cancer development and treatment. From birth and throughout whole life, the human beings keep an intimate mutually beneficial relationship with the microbial community, using it as protection against external aggressions and as a supplier of beneficiary essential molecules.

The human microbiota is the ecological community of commensal, symbiotic and pathogenic microorganisms, including protozoa, fungi, bacteria, and viruses. Microbiota is acquired after birth by vertical transmission inheritance from the mother through direct surface contact, beginning shortly after that its evolution (in response to environmental factors, diet, drugs or environmental exposures) cumulating in a stable adult microbiota by the age of two (18). Most studies have been focused on the bacterial composition of microbiota, since these microorganisms are the most abundant within commensal microbiota. Indeed, it is estimated that a single human subject harbors more than one billion bacterial cells, most of which are commensal, so the total microbiota is estimated to constitute 0.2 kg of weight (19).

The size and the composition of the microbiota differ by anatomical site, showing a wide interpersonal variation but relatively temporal stability in a single individual (20). Thus, the individual composition of microbiota should be taken into account when using it as a biomarker for cancer treatment as well as to design effective microbiota transplant protocols, which are major current challenges in different pathologies including lung cancer, as will be discussed in the next sections.

As indicated above, among other functions, microbiota regulates host immunity and tissue homeostasis. Therefore, the same microorganisms that are beneficial to human health, under certain circumstances, can promote the development of diseases and cancer (21–25). The eubiosis is conditioned by multiple factors: hereditary and environmental factors, genetic background, diseases, lifestyle (mainly diet), chronic infections or antibiotic exposure, among others (26). These factors can contribute to the perturbation of the balance of microbiota composition, a situation known as dysbiosis. This situation can be mild and temporal reverting after the detrimental stimulus is removed. However, in some situations, this dysbalance can be chronified, altering tissue homeostasis and leading to diseases like cancer (25). Direct effects of microbial components on cell transformation or indirect effects related with a dysregulated inflammatory immune response have been found to be involved in carcinogenesis (27).

## Lung Microbiota, Cancer and Immunotherapy

The gut microbiota gives the major contribution to human microbiota and comprises about 3 × 10^13^ bacterial cells, followed by the skin, which is estimated to harbor ~10^12^ bacteria (28). Accordingly, the intestinal microbiota and its role in regulating host metabolism and gastrointestinal and metabolic diseases such as gastric cancer (29, 30), inflammatory bowel disease (a risk factor for colorectal cancer), diabetes, and obesity (31–33) is the most studied so far. However, recent findings indicate that other microbial populations in the body like lungs are very important in keeping tissue homeostasis and contribute to diseases like asthma, COPD or lung cancer (27).

Lungs have traditionally been considered sterile space, nevertheless they are constantly exposed to microorganisms since the lower respiratory tract is replete with diverse communities of bacteria both in health and in diseased states (34). Bacterial communities in the lungs are dynamic as the airways are constantly exposed to microorganisms suspended in the air that flow through the oral cavity and upper respiratory tract to the lower respiratory tract. The composition of lung microbiota modulates a healthy host lung immune system. A proper balance between lung immunity and microbiota is key to fight infection but, in addition, to prevent lung inflammatory diseases like cancer. The epithelial cell barrier is the first line of defense in lungs preventing invasion by pathogenic microbes and, at the same time, establishing key molecular interactions with the commensal microbiota that regulates immune homeostasis. Commensal microbiota regulates immune tolerance, decreasing lung inflammation through dendritic cell (DC), γδ T, and T regulatory (Treg) cell recruitment (27). Other resident immune cells like macrophages, Innate Lymphoid Cells (ILCs) and T cell subsets contribute to regulate local inflammatory responses by preventing the overload of pathogens or their metabolites. Lung microbiota dysbiosis can contribute to cancer development by inducing a dysregulated immune response characterized by an overactivation of inflammatory cells like M1 macrophages, Th1 cells or γδ T cells. Inflammation contributes to cell transformation and cancer by activating cell proliferation pathways as well as by inducing radical oxygen species and genetic mutations. The specific connection between airway microbiota, immune regulation and lung cancer is not clear yet. As an example it was recently shown that the presence of a lung bacterial composition enriched in oral commensals is associated with a prevalence of T helper 17 (Th17) inflammation that seems to be key for a proper regulation of the lung immune response (35). However, the connection between Th17 cells, chronic lung inflammation and cancer is not yet clear.

Although less explored than the connection between microbiota dysbiosis, chronic inflammation and cancer development, microbial components can contribute to lung cancer development by producing metabolites with oncogenic potential. For example, Tsay et al. (36) found that exposure to *Veillonella, Prevotella*, and *Streptococcus* bacteria induce epithelial cell transformation *in vitro* and *in vivo* by activating the PI3K and ERK pathways.

The respiratory microbiome differs significantly between the three major anatomical regions: oral/nasal, upper and lower respiratory tract (37). Taking into account the communication between the oral cavity and the respiratory tract, the oral microbiome could be highly relevant to the lung microbiome. Nevertheless, not every alteration observed in sputum represents changes in the microbiome of the lower airway microbiome, since there is increasing evidence that the microbiome of sputum is a better reflection of the oral microbiome than of the lower airway microbiome (38–40).

Movement of microbes between these sites and the lungs occurs regularly via breathing and microaspiration of pharyngeal secretions, which seems to be the main source of lung microbiota in healthy subjects (41). Despite this connection, it should be considered that the composition and function of upper and lower airways microbiome are not identical and (37), thus, these differences should be had in mind to design studies that can be successfully applied to the diagnosis and treatment of cancer. For example, identification of microbiome composition in oral cavity or saliva could be useful to identify biomarkers to predict ICI response, but when designing interventions to modulate lung microbiota composition and enhance ICIs efficacy, genuine identification of lung microbiota might be required.

Several sample types are used to analyzed lung microbiome composition: bronchoalveolar lavage (BAL), bronchial brushing tissue, buccal sample, surgical resection tissue or exhaled breath condensate (23, 42), among others. Large number of samples, use of non-invasive studies, and exact statistical analysis are crucial for detection of predominant bacterial species in lung cancer (43). Because of the mentioned technical reasons, characterization of the oral microbiome is less complex than characterization of lung microbiome. The oral microbiota includes bacteria, fungi, as well as viruses. It has been associated with systemic diseases including infective endocarditis, rheumatoid arthritis, pulmonary disease, and in more recent studies, with cancer (44–46). It is important to take into account that the oral microbiota can aid in conversion of alcohol and smoking byproducts to mutagenic compounds, so that the oral dysbiosis may be related to premalignant processes and the cancer itself (34). Thus, analyses of oral microbiota might also be interesting to find out predictive biomarkers for ICI or even to modulate its composition to prevent cancer development or progression or to enhance ICI efficacy.

The number of studies in the field of lung microbiota is rapidly growing and have yielded provocative observations highlighting a possible relationship between lung microbiota and respiratory disease (23). The development of lung cancer seems to be closely associated with chronic inflammation characterized by infiltration of inflammatory cells and accumulation of pro-inflammatory factors (cytokines, chemokines, and prostaglandins), a high bacterial load and linked to airway dysbiosis or reduction in the dominant bacterial species in the microbiota (37, 47–49).

Specific microbes have been associated with lung cancer (*Pseudomona, Veillonella, Streptococcus, Provotella, Fusobacteria…*) (50, 51) as *Bacteroides, Firmicutes*, and *Proteobacteria* are the most common phyla consistently observed in lung microbiota of healthy individuals (23, 52). Higher richness and diversity in normal tissue seems to be significantly associated with reduced recurrence-free and disease-free survival. Family Lachnospiraceae, genera *Faecalibacterium* and *Ruminococcus*, among others, are associated with reduced recurrence-free and disease-free survival (53).

Paramount to the development of microbiota-based therapeutics, the next challenge in microbiome research will be to identify individual microbial species that causally affect cancer phenotypes and unravel the underlying mechanisms (54). Metagenomics studies using culture-independent techniques such as PCR amplification and sequencing of the 16S ribosomal (r)RNA gene, especially variable regions V3-V4 of 16S rRNA bacterial gene sequencing, enables the identification and classification of the species without requiring pre-cultivation (25, 55). The sequencing of basal stool and saliva samples from patients treated with ICIs has obtained striking preliminary results that suggest a close relationship between the commensal microbiome and the clinical efficacy of immunotherapy, which might be useful to stratify patients based on their metagenomic microbial fingerprint (28). However, it should be taken into account that rRNA sequencing has limitations since it cannot differentiate between living and dead microbes. Thus, metagenomic analyses just provide the picture of the microbial composition (microbiome) without providing information on the potential functional consequences of those microbes. Here the analyses of genes expressed by the microbial community (metatranscriptomics) and their byproducts (metabolomics) can be very useful since they provide information on the functional state of microbiota and the potential consequences for the health of the environmental niche they inhabit, the lung in this case. Thus, the combination of metagenomics, metatranscriptomics, and metabolomics will provide a whole picture on the composition of lung microbiota and their functional consequences under different conditions (56).

ICIs act by blocking pathways of negative regulation of the immune system, in order to enhance antitumor immune response. Due to the general dysregulation of immune homeostasis, ICIs induce a broad spectrum of side effects potentially involving any organ, known as immune-related adverse events (irAEs) (57). irAEs occur more commonly in patients on anti-CTLA- 4 treatment as compared with those taking anti-PD-1/-L1, and the incidence of irAEs increases when these agents are used in combination. It has been reported that patients who receive ICIs and have immuno-related toxicity (58, 59) have a different intestinal microbiota composition, in addition to a possible greater benefit of treatment (60, 61). Thus, further studies correlating these factors are of interest in order to establish microbiome composition as a predictive biomarker for ICI efficacy and toxicity.

There are multiple studies exploring the possibility that dysbiosis associated with malignant disease or concomitant antibiotic use (before, during or shortly after treatment with ICIs) could influence ICI efficacy. NSCLC patients taking antibiotic during ICI treatment have a significant reduction in progression free survival and overall survival compared with those who did not receive antibiotics (62). Here it should be taken into consideration that antibiotic use might be associated with a more severe disease and thus, this might act as a confounding factor when studying the correlation between antibiotic and ICI efficacy. Although most studies have focused on the intestinal microbiota, it is expected that antibiotics could also influence the lung microbiota, altering the antitumoral effect of ICIs, anti-CTLA4 and anti-PD-1 / L1 in patients with lung cancer (63).

## Mucosal Barrier Integrity and Microbiota

Gut and lung microbiota are connected thanks to a complex bidirectional axis via lymphatic and blood circulation, which may explain why changes in one mucosal compartment could directly impact a distant mucosal site (64).

Gut microbes can influence lung immune function through different mechanisms. One would be the connection through molecular patterns associated with pathogens, such as lipopolysaccharides (LPS), which can stimulate toll-like receptors and activate genes that regulate inflammation and responses of the innate immune system. In addition, they would cause phenotypic changes in dendritic cells (DCs) and migration to mesenteric lymphoid nodes to promote the priming of T lymphocytes and the production of various regulatory cytokines (IL-10, TGF-β, IFNγ, and IL-6). In these lymph nodes, T cells acquire localization molecules that initiate migration to the respiratory mucosa, through CCR4 or CCR6, where they promote protective and anti-inflammatory responses. Another mechanism would be metabolites such as short chain fatty acids (SCFA), produced by the fermentation of carbohydrates by bacteria, which modify gene expression through the inhibition of histone deacetylases, methylation, the production of cytokines and chemokines, and cell differentiation, proliferation and apoptosis (18, 65–67).

Intestinal dysbiosis leads to perturbation of gut immune homeostasis favoring the development of inflammatory processes and even tumoral processes (30, 57). The permeability of the epithelial barriers, like intestinal and respiratory tract, is closely related to the luminal content and the interaction with the microbiota. Permeability depends on the junctional complex between the intestinal enterocytes, which includes tight junction (TJ) proteins that regulate the transport of ions and water between the gut lumen and the blood stream. Increased gut permeability causes the trespass of antigens to the lamina propia and the blood stream, promoting a local and systemic inflammatory immune responses enhancing epithelial barrier loss and affecting distant organs and tissues (68). The protein zonuline is an admissible regulator of TJs permeability and the only known physiological regulator of intestinal TJs, and its presence in serum has been proposed as a peripheral marker to assess gut permeability (69), as well as endotoxin. (70, 71). Thus, the loss of barrier function may be suspected by a positive zonuline regulation (68, 72–74). However, there are many unknowns about the role of these components, their relationship with the microbiota, with the use of antibiotics and with the response to immunotherapy. In addition, it has been recently shown that detection of zonuline in serum might be affected by serious technical flaws and thus its value to monitor epithelial barrier permeability should be confirmed (75).

On the other hand, plasma citrulline, an amino acid produced by enterocytes, has been validated as a marker of the intestinal barrier and function of enterocytes. Citrulline can be produced as a result of a post-transduction modification from arginine in a reaction catalyzed by peptidyl-arginine deaminase. This modification occurs in inflammatory processes and cell death. In these processes, citrulline can be recognized aberrantly by the immune system, leading to the generation of anti-citrulline antibodies. Under some circumstances, such as infections and the need for antibiotic treatment in patients with lung cancer undergoing treatment with ICIs, inflammatory phenomena and cell death may occur leading to aberrant recognition of citrulline and the generation of anti-citrulline antibodies. Finally, it seems that citrulline levels are affected with the use of antibiotics and can be correlated with the response to immunotherapy (76). Identification of soluble circulating factors that might be markers of the integrity of the epithelial barrier could be very useful to monitor cancer progression and treatment efficacy as discussed below.

Several studies have studied the correlation between gut microbiome and outcomes to ICIs. A greater diversity of the intestinal microbiome seems to be related to a greater progression-free survival (77), showing a higher concentration of *Akkermansia muciniphilia* in the stools of the responders (62).

## Challenges and Future Perspectives

Understanding how microorganisms present in the respiratory tract can influence the development of lung carcinoma and the effectiveness of treatments is key to develop therapeutic approaches to modify the microbial composition and thus improve their effectiveness.

Future precision medicine strategies will likely be based on new diagnostic and therapeutic tools that identify alterations in the microbiome in each patient that could be used as new biomarkers for individual treatments (28). These biomarkers could also help identify candidate patients for microbiota intervention strategies such as fecal microbiota transplantation (FMT) with the possibility of evaluating the effects of selected members of the commensal gut microbiota on systemic immunity, including in the lungs, as well as the use of probiotics and prebiotics (78, 79). FMT, usually used to treat recurrent *Clostridium difficile* infection, could be used as therapy to treat other diseases related to an unhealthy gut microbiota. However, before implementing these approaches it should be clarified how to prepare patients for FMT (preconditioning) and how to manage possible complications related to antibiotic resistance and the potential of infections due to FMT.

The modulation of microbiota is considered a relevant target for novel therapeutic and preventive treatments against a range of diseases (70), albeit this field is still in its infancy and more research is required before microbiota can be safely manipulated to prevent or treat disease. The microbiome may be potentially manipulated with an aim to correct dysbiosis through nutritional interventions such us use of probiotics, prebiotics or antibiotics, among others, seeking for a reversion of immunosuppression present in the tumor microenvironment (80). For example it has been found that oral administration of *Bifidobacterium* induces dendritic cell function and increases CD8+ T cell accumulation in the tumor microenvironment, improving response to anti-PDL1 antibody in mouse models of cancer (81). Despite some advances, caution is warranted as the ideal beneficial lung microbiota and how to change it as well as the potential risks of this modification are not yet known. For example, uncontrolled microbiota manipulation might induce detrimental inflammatory reactions that could worsen rather than improve cancer evolution and treatment efficacy.

Several studies have supported that the lung microbiota may play a key role in carcinogenesis and in response to chemotherapy and immunotherapy. Gut microbiome is the most studied so far. Lung microbiome is currently being studied to determine how and when does it affect lung cancer development and the best techniques to analyze it. New techniques based on microbiota-associated metabolic fingerprints can be useful to identify microbiota composition as recently developed for gut microbiota from lung cancer patients without requiring RNA analyses (82). Notably, it has been found that the metabolic profile in lungs from diseased patients, including lung cancer, significantly differs from that in healthy individuals (83–85). Notably some studies have found that specific metabolites are enriched in respiratory airways of patients with different diseases and this profile correlates with the presence of specific bacterial species (86–88).

These results indicate that characterization of lung metabolome could be used as a surrogate marker of changes in lung microbiota in order to provide an easier and more robust technology to use lung microbiota (or their products) as a biomarker for diagnosis and immunotherapy response in lung cancer. A recent study found that the presence of specific microbial metabolites in plasma correlated with anti-PD1 efficacy in lung cancer patients (89). Although similar studies have not been performed yet in airways, a recent study has shown that the profile of Volatile Organic Compounds in exhaled breath from lung cancer patients determined using an electronic nose predicts the efficacy of anti-PD1 treatment (90). Although future studies will be required to characterize the origin of these metabolites (host and/or microbial) and its correlation with changes in airway microbial composition and the response to anti-PD1 therapy, this approach would be very useful to implement the use of airway microbiota in clinical management of lung cancer and immunotherapy treatment.

Analysis of the upper respiratory tract microbiota as the source of the lung and gastric microbiota could be useful if the correlation between both (oral/upper respiratory tract and lung/gut) would be demonstrated. The sampling would be facilitated by less invasive techniques for the patient (nasal smears, saliva samples.), with the possibility of evaluating the changes over time.

The study of the microbiota is a field that has just made its way, offering opportunities and challenges for improving immunotherapy efficacy. Our group is conducting a pilot study in this direction in lung cancer patients receiving immunotherapy.

## Outstanding Questions

1. Can we use antibiotics to safely modulate the microbiota? How can we modify resistant bacteria's composition and achieve the proper balance? What are the risks of microbiota manipulation?
2. What conditioning does a patient require prior to a fecal microbiota transplant? What is the proper composition for each individual?
3. Would it be useful to have an individualized microbiota bank?
4. Which barrier is primarily responsible for the loss of epithelial barrier in lung cancer? Are there specific markers that allow us to differentiate them?

## Author Contributions

MO-D conceived the paper. MO-D, EG, DI, and JP draft the first version of the manuscript. MC, MG, RL, LM-L, AR-L, JRP, AS, IT, and AY commented and added extra information. All authors reviewed and approved the final version.

## Conflict of Interest

The authors declare that the research was conducted in the absence of any commercial or financial relationships that could be construed as a potential conflict of interest.
